# Effect of Lifestyle Changes after Percutaneous Coronary Intervention on Revascularization

**DOI:** 10.1155/2020/2479652

**Published:** 2020-02-14

**Authors:** Yang Wang, Ying Xian, Tao Chen, Yanyan Zhao, Jinggang Yang, Bo Xu, Wei Li

**Affiliations:** ^1^Medical Research & Biometrics Center, National Center for Cardiovascular Diseases, Fu Wai Hospital, Chinese Academy of Medical Sciences and Peking Union Medical College, Beijing, China; ^2^Duke Clinical Research Institute, Durham, NC, USA; ^3^Tropical Clinical Trials Unit, Department of Clinical Sciences, Liverpool School of Tropic Medicine, Liverpool, UK; ^4^Fu Wai Hospital, National Center for Cardiovascular Diseases, Chinese Academy of Medical Sciences and Peking Union Medical College, Beijing, China

## Abstract

**Objective:**

Whether optimal cardiovascular health metrics may reduce the risk of cardiovascular events in secondary prevention is uncertain. The study was conducted to evaluate the influence of lifestyle changes on clinical outcomes among the subjects underwent percutaneous coronary intervention (PCI).

**Methods:**

The study group consists of 17,099 consecutive PCI patients. We recorded data on subject lifestyle behavior changes after their procedure. Patients were categorized as ideal, intermediate, or poor CV health according to a modified Life's Simple 7 score (on body mass, smoking, physical activity, diet, cholesterol, blood pressure, and glucose). Multivariable COX regression was used to evaluate the association between CV health and revascularization event. We also tested the impact of cumulative cardiovascular health score on reoccurrence of cardiovascular event.

**Results:**

During a 3-year median follow-up, 1,583 revascularization events were identified. The observed revascularization rate was 8.0%, 9.3%, and 10.6% in the group of patients with optimal (a modified Life's Simple 7 score of 11–14), average (score = 9 or 10), or inadequate (less or equal than 8) CV health, respectively. After multivariable analysis, the adjusted hazard ratios were 0.83 (95% CI: 0.73–0.94) and 0.89 (95% CI: 0.79–0.99) for patients with optimal and average lifestyle changes comparing with the inadequate tertile (*P* for trend = 0.003). In addition, each unit increase in above metrics was associated with a decrease risk of revascularization (HR, 0.96; 95% confidence interval, 0.93–0.98; *P* for trend = 0.003). In addition, each unit increase in above metrics was associated with a decrease risk of revascularization (HR, 0.96; 95% confidence interval, 0.93–0.98;

**Conclusion:**

Ideal CV health related to lower incidence of cardiovascular events, even after the percutaneous coronary intervention. Revascularization can be reduced by lifestyle changes. The cardiovascular health metrics could be extrapolated to secondary prevention and need for further validation.

## 1. Introduction

Ideal cardiovascular health (CVH) has been proposed by the American Heart Association (AHA) and used to measure population health [1]. The seven risk factors (Life's Simple 7) that people can improve through lifestyle changes included four health behaviors (stop smoking, eat better, get active, and lose weight) and three health factors (manage blood pressure, control cholesterol, and reduce blood sugar). Cumulative evidence already demonstrated the AHA ideal CVH metrics could be used for cardiovascular health factors assessment, health promotion, and a tool to predict mortality and cardiovascular diseases (CVD) risk [2, 3]. The steep gradient relationship between ideal CVH metrics and CVD was similar across different regions and diverse race-ethnic groups [4–8].

The concept of ideal CVH metrics was originally defined and intended to use for primordial prevention among general population [1, 9]. Although the inverse relationship between ideal CVH and CVD incidence was also well documented for primary prevention [10–15], the evidence in secondary prevention is limited [16, 17]. It should be noticed that most of the individual components in ideal CVH metrics associated with reduced clinical event risk for the subject with established CVD [18, 19]. However, few data are available on the relationship between having ideal risk factor profile using a composite measure and the recurrence of cardiovascular events.

Therefore, our aim in this study is to investigate the influence of ideal CVH as a risk factor of cardiovascular outcomes for secondary prevention. The study was based on a cohort of patients who underwent percutaneous coronary intervention. We hypothesized that the subjects with optimal CVH would be less likely to develop cardiovascular events during their follow-up period.

## 2. Methods

### 2.1. Study Design and Population

The current analysis was based on an established cohort from Fuwai hospital. A total of 19,506 consecutive patients with successful percutaneous coronary intervention were recruited. Further inclusion criteria for analysis were as follows: the subject should have at least one stent implantation, one year or longer postprocedure follow-up, alive, complete the questionnaire during follow-up visit. Finally, there were 17,099 (87.7%) patients fulfill the above requirements. The study protocol was approved by ethical committee, and formal inform consent was obtained from every study participants. Details of the study design have been previously described [20].

### 2.2. Follow-Up

Follow-up was conduct by a group of trained investigators. The standard operation procedure was fixed after a small scale pilot study. The nonresponder was the subject who cannot be reached after 3 contacts on different days within one week. Both information on lifestyle changes and clinical outcomes were collected in a standardized questionnaire. A 5% random resampling process was carried out to validate the reliability of the data collected by the above interview procedure (kappa coefficients were from 0.91 to 0.97 for different items in the questionnaire).

### 2.3. Exposure and Outcome

Prespecified options (exp. greater, no change, or less) had been used to reflect the lifestyle behavior changes after PCI procedure compared with the situation before procedure. A modified Life's Simple 7 score (on body mass, smoking, physical activity, diet, cholesterol, blood pressure, and glucose) had been developed according to AHA recommendation (giving 2 points for ideal, 1 point for intermediate, and 0 point for poor). For physical activity, 2 = longer, 1 = no change, and 0 = shorter. The blood pressure, cholesterol, and glucose were used the same rule: 2 = better controlled than before, 1 = no change, and 0 = worsen. Healthy diet covered fresh vegetables/fruits, salt, and meat. If a patient reported more fresh vegetables/fruits, less salt, and meat consumption, the score for healthy diet was 2. On the opposite, if a patient had less vegetables/fruits, more salt, or meat compare with before procedure status, the healthy diet score was 0. The remained situations were assigned 1 for the diet score. For weight changes, 2 = no change, 1 = loss weight, and 0 = weight increase. If a patient was a nonsmoker or they quit smoking at least 1 year before their procedure, the nonsmoking score was 2. For smokers and other former smokers, the nonsmoking score were 0 and 1, respectively. After obtaining the modified Life's Simple 7 score, both the cumulative score (ranged from 0 to 14) and its tertiles (1^st^ tertile: inadequate CVH, 2^nd^ tertile: average CVH, and 3^rd^ tertile: optimal CVH) were used to estimate the impact on reoccurrence of cardiovascular event. The key clinical outcome in current analysis was any revascularization during the follow-up period.

### 2.4. Statistical Analysis

Means and standard deviations were used as descriptive analysis for continuous variables. Categorical variables used frequencies and proportions. The patients were divided into 3 groups according to their tertiles of the modified Life's Simple 7 score. The one-way ANOVA or Chi-square test was used for between groups comparison where appropriate. To evaluate the potential association between the modified Life's Simple 7 score and revascularization, the univariable and multivariable COX regression model had been used. The covariates were fixed according to the published literature (included demographic, health status, family health history, and procedure related characteristics). Firstly, the trend between each ideal CVH group had been tested. After that, dummy variables were used to represent the patient with an optimal (11–14) and average (score = 9 or 10) modified Life's Simple 7 score and the lowest tertile (inadequate: the score less than or equal to 8) group was used as reference. In addition, the risk of revascularization for each unit increase in ideal CVH metrics was estimated under the same confounding variables adjustment model. The analysis software was SAS®9.4, and significant level in this study was 2-sided 0.05.

## 3. Results

### 3.1. Characteristics of Study Population

A total of 17,099 percutaneous coronary intervention patients (78.7% male) with a mean age of years 57.5 ± 10.4 were enrolled in this analysis. Two-thirds of the patients were diagnosed as unstable ungina. The proportion of hypertension, dyslipidemia, and diabetes among the overall population was 50.0%, 32.0%, and 18.5%, respectively. A total of 1,583 revascularization events during the follow-up period had been identified. The participants were grouped by the occurrence of revascularization (Yes/No). Detailed demographic characteristics are listed in Table 1.

### 3.2. Lifestyle Behavior (Ideal Cardiovascular Health Metrics) Changes

Table 2 shows the prevalence of each component of cardiovascular health metrics. Most subjects (72.8%) could manage and maintain weight at appropriate range after their PCI procedure. More than half of the patients were nonsmoker or permanent quit smoking. Over forty percent subjects moved to a healthy diet behavior after their discharge from hospital. A better control of blood pressure, cholesterol, and blood glucose was achieved in 59.5%, 55.7%, and 22.1% among the overall participants, respectively. The proportions of each individual ideal CVH component among the tertile groups (determined by the cumulative score: inadequate, average, and optimal) had also been described.

### 3.3. Uni- and Multivariable COX Regression Analysis

Firstly, we simply counted the cumulative score of ideal Life's Simple 7 components. The hazard ratio of 1 unit change on the ideal CVH metrics was 0.96 (95% CI, 0.93 to 0.98) after the adjustment of potential confounding variables. The multivariable COX regression model shows, comparing with the inadequate category (the lowest tertile on lifestyle behavior modification), the hazard ratios on revascularization for patients in the average and optimal ideal CVH group were 0.89 (95% CI, 0.79 to 0.99) and 0.83 (95% CI, 0.73 to 0.94), respectively. The *P* for the trend was 0.003. The relationships between each individual ideal CVH component and repeated revascularization event were ranged from 0.79 to 1.05 (hazard ratios by the multivariable adjusted model). Detailed results are described in Table 3.

## 4. Discussion

### 4.1. Key Findings and Study Strengths

Our study suggested subjects in optimal ranges of Life's Simple 7 (LS7) had a lower risk of revascularization compared with people in poor ranges during a 3 year follow-up period after percutaneous coronary intervention. Each additional ideal cardiovascular health metric was associated with 4% lower risks of repeated revascularization event. To the best of our knowledge, this study is the first to investigate the association of ideal cardiovascular health metrics with clinical outcomes among participants underwent percutaneous coronary intervention.

### 4.2. Comparisons with the Published Literature

Current percutaneous coronary intervention studies are more focused on examining the efficacy of different treatment strategies of emerging or existing devices and of the value of coronary physiology or intravascular imaging in PCI planning [21]. Evidence regarding the links between healthy lifestyle and cardiometabolic consequences in people who had coronary interventions is fairly sparse. Potential benefit of Life's Simple 7 had been investigated among myocardial infarction patients. The findings suggested ideal CV health at middle age was associated with better prognosis after MI in later life [22]. However, the impact of ideal cardiovascular health metrics among subjects with different risk strata has not been well established. In a recent large scale nationwide prospective cohort study, participants with prediabetes or diabetes who had five or more ICVHMs (ideal cardiovascular health metrics) exhibited lower or no significant excess risk of CVD events compared with those with normal glucose regulation. Compared with 1 ideal CVH metric or none, 5 or more ideal metrics were associated with 58% and 61% lower CVD risks among participants with prediabetes and diabetes, respectively [23]. The attenuated effect size observed in our study may attribute to the heterogeneity between different populations. Our study extends previous findings by comprehensively assessing 7 lifestyle risk factors in secondary prevention for revascularization in relation to lifestyle factors individually and in combination.

Baseline measurement of ideal CVH and the longitudinal maintenance of CVH were both significant associated with CVD progression in general population [24, 25]. However, it should be noticed the prevalence of ideal cardiovascular health metrics was systematic different in secondary prevention [26]. For example, the prevalence of smoking in general population has been reported to be 52.9% [27]. However, the proportion for quit smoking was only 8% [28]. In contrast, the smoking cessation rate was 40% to 94% at 1 year and 37% at 5 years after the ischemic event [29, 30]. Further, a pooled cohorts consisted of 661,137 participants indicate a benefit threshold at approximately 3 to 5 times the recommended leisure time physical activity [31]. Around one fifth participants could meet the above intensity of physical activity for general population. Compare with our study, the observed proportion of patient had increased level of physical activities after the PCI procedure was around thirty percent (27.7%). Cardiovascular intervention is an opportunity to reassess the risk factor control and an optimal time when patients and family members are more likely to be receptive to lifestyle modification [32]. Healthcare professionals should encourage PCI patients to perform more ideal CVH metrics.

Although cardiac rehabilitation is strongly recommended following myocardial infarction, which components of rehabilitation are most beneficial is unclear [33]. As one key component of cardiovascular health metrics, smoking increases the risk of virtually all cardiovascular disease subtypes [34]. Smoking cessation had been demonstrated as a modifiable risk factor both for primary and secondary prevention of stroke [35–37]. However, the effect of single ideal CVH metrics maybe partly attributable to other lifestyle behavior changes (eg., the subject has more exercise and healthier diet at the same time with smoking cessation) [36, 38]. The combination of cardiovascular health metrics may have a joint impact on the endothelialization and inflammatory process. This proposed phenomenon was corresponding to the underling mechanism of restenosis of the coronary arteries [39, 40]. Further basic researches are required to validate the above hypothesis.

### 4.3. Study Limitations

Our study has several limitations. First, we could not fully rule out all the residual and unmeasured confounders, such as genetic predisposition, medications, psychological status, and possible reverse causation. Nevertheless, the sensitivity analysis taking into account this potential bias showed similar results. Second, the cardiovascular health metrics were modified according to the feature of the follow-up process in this study. The changes in the metrics over time (health check-up periods) could not be accounted for in this study. Furthermore, participants were excluded if their cardiovascular health metrics are missing, so the selection bias may also exist. Third, measurement errors in self-reported assessments of lifestyle changes were inevitable, although the accuracy of self-reported information had been demonstrated through a 5% resampling validation process. The use of prospectively collected, cumulatively averaged values based on repeated assessments would reduce the effect of random measurement error. Due to the aforementioned reasons, our results should be interpreted cautiously.

## 5. Conclusions

In this observational study, patients underwent percutaneous coronary intervention who achieved a greater number of ideal CVH metrics exhibited lower risk of repeated revascularization event. Our findings emphasize the importance of promoting the adherence to ideal CVH metrics in the population with established cardiovascular disease. We believe further researches addressing this hypothesis are warranted.

## Figures and Tables

**Table 1:** Baseline characteristics of participant with or without revascularization.

Variables	Revas. (*N* = 1583)	No revas. (*N* = 15516)	*P* value
Age, *y*, mean ± SD	58.1 ± 10.4	57.4 ± 10.4	0.023
Male, *n* (%)	1259 (79.5)	12201 (78.6)	0.406
Unstable angina, *n* (%)	1028 (64.9)	10228 (65.9)	<0.001
Prior myocardial infarction, *n* (%)	570 (36.0)	4755 (30.7)	<0.001
Family history of CHD, *n* (%)	88 (5.6)	611 (3.9)	0.002
Hypertension, *n* (%)	911 (57.6)	7636 (49.2)	<0.001
Dyslipidemia, *n* (%)	574 (36.3)	4905 (31.6)	<0.001
Diabetes, *n* (%)	349 (22.1)	2821 (18.2)	<0.001
LVEF < 40%, *n* (%)	683 (43.2)	8516 (54.9)	<0.001
Reference vessel diameter, mm, mean ± SD	3.1 ± 0.6	3.2 ± 1.9	<0.001
Lesion length, mm, mean ± SD	26.1 ± 15.7	25.4 ± 14.6	0.078
Diameter stenosis, %, mean ± SD	89.7 ± 7.7	88.4 ± 8.0	<0.001
Calcification, *n* (%)	72 (4.6)	534 (3.4)	0.023
Total occlusion, *n* (%)	469 (29.6)	3231 (20.8)	<0.001
Transradial access, *n* (%)	1140 (72.0)	12495 (80.5)	<0.001
TIMI classification, *n* (%)			<0.001
0	421 (26.6)	3232 (20.8)	
1	75 (4.7)	648 (4.2)	
2	202 (12.8)	1945 (12.5)	
3	885 (55.9)	9691 (62.5)	

**Table 2:** Prevalence of ideal cardiovascular health metrics.

	Overall (*N* = 17099)	Inadequate (*N* = 5267)	Average (*N* = 6029)	Optimal (*N* = 5803)
Physical activity				
Poor	2785 (16.3)	1521 (28.9)	907 (15.0)	357 2.8)
Intermediate	9674 (56.0)	3254 (61.8)	3775 (62.6)	2545 (26.6)
Ideal	4740 (27.7)	492 (9.3)	1347 (22.3)	2901 (61.2)

Blood pressure				
Poor	1290 (7.5)	1000 (19.0)	250 (4.2)	40 (0.7)
Intermediate	5642 (33.0)	3150 (59.8)	2179 (36.1)	313 (5.4)
Ideal	10167 (59.5)	1117 (21.2)	3600 (59.7)	5450 (93.9)

Blood cholesterol				
Poor	1371 (8.0)	1003 (19.0)	315 (5.2)	53 (0.9)
Intermediate	6205 (36.3)	3348 (63.6)	2446 (40.6)	411 (7.1)
Ideal	9523 (55.7)	916 (17.4)	3268 (54.2)	5339 (92.0)

Blood glucose				
Poor	1977 (11.6)	1179 (22.4)	621 (10.3)	177 (3.1)
Intermediate	11337 (66.3)	3751 (71.2)	4525 (75.1)	3061 (52.8)
Ideal	3785 (22.1)	337 (6.4)	883 (14.7)	2565 (44.2)

Ideal BMI				
Poor	2046 (12.0)	1098 (20.9)	700 (11.6)	248 (4.3)
Intermediate	2599 (15.2)	1027 (19.5)	877 (14.6)	695 (12.0)
Ideal	12454 (72.8)	3142 (58.7)	4452 (73.8)	4860 (83.6)

Healthy diet				
Poor	818 (4.8)	543 (10.3)	207 (3.4)	68 (1.2)
Intermediate	9035 (52.8)	3720 (70.6)	3592 (59.6)	1723 (29.7)
Ideal	7246 (42.4)	1004 (19.1)	2230 (37.0)	4012 (69.1)

Ideal smoking status				
Poor	3683 (21.5)	2003 (38.0)	1190 (19.7)	490 (8.4)
Intermediate	4156 (24.3)	1557 (29.6)	1420 (23.6)	1179 (20.3)
Ideal	9260 (54.2)	1707 (32.4)	3419 (56.7)	4134 (71.2)

**Table 3:** Hazard ration (95% CI) of revascularization according to combined and individual ideal CVH metrics.

	Univariable analysis	Multivariable analysis
Combined ideal CVH metrics		
1 unit change (each 1-number increment in ICVHMs)	0.95 (0.93, 0.98)	0.96 (0.93, 0.98)
1 tertile change (*P* for trend)	0.90 (0.85, 0.96) <0.001	0.91 (0.85, 0.97) 0.003
Average vs. Inadequate	0.88 (0.79, 0.99)	0.89 (0.79, 0.99)
Optimal vs. Inadequate	0.81 (0.72, 0.92)	0.83 (0.73, 0.94)

Individual component of ideal CVH metrics—Physical activity		
1 unit change	0.92 (0.85, 0.99)	0.92 (0.85, 0.99)
*P* for trend	0.038	0.037

Individual component of ideal CVH metrics—blood pressure		
1 unit change	0.89 (0.82, 0.96)	0.90 (0.84, 0.97)
*P* for trend	0.002	0.008

Individual component of ideal CVH metrics—blood cholesterol		
1 unit change	0.88 (0.82, 0.95)	0.89 (0.83, 0.96)
*P* for trend	0.001	0.004

Individual component of ideal CVH metrics—blood glucose		
1 unit change	0.79 (0.72, 0.86)	0.79 (0.72, 0.86)
*P* for trend	<0.001	<0.001

Individual component of ideal CVH metrics—ideal BMI		
1 unit change	0.95 (0.89, 1.02)	0.94 (0.88, 1.01)
*P* for trend	0.192	0.110

Individual component of ideal CVH metrics—healthy diet		
1 unit change	0.98 (0.90, 1.06)	1.01 (0.92, 1.10)
*P* for trend	0.586	0.885

Individual component of ideal CVH metrics—ideal smoking status		
1 unit change	1.05 (0.98, 1.11)	1.05 (0.98, 1.12)
*P* for trend	0.153	0.150

## Data Availability

The data that support the findings of this study are available from the corresponding author upon reasonable request.
